# Adaptive Indoor Positioning Model Based on WLAN-Fingerprinting for Dynamic and Multi-Floor Environments

**DOI:** 10.3390/s17081789

**Published:** 2017-08-05

**Authors:** Iyad Husni Alshami, Noor Azurati Ahmad, Shamsul Sahibuddin, Firdaus Firdaus

**Affiliations:** 1 Department of Computer Science, Islamic University of Gaza, Gaza, Palestine; 2 Advanced Informatics School (AIS), Universiti Teknologi Malaysia, Kuala Lumpur 54100, Malaysia; shamsul@utm.my; 3 Department of Electrical Engineering, Islamic University of Indonesia, Yogyakarta 55584, Indonesia

**Keywords:** indoor positioning, fingerprint, WLAN, RSSI, multi-floor, path loss model

## Abstract

The Global Positioning System demonstrates the significance of Location Based Services but it cannot be used indoors due to the lack of line of sight between satellites and receivers. Indoor Positioning Systems are needed to provide indoor Location Based Services. Wireless LAN fingerprints are one of the best choices for Indoor Positioning Systems because of their low cost, and high accuracy, however they have many drawbacks: creating radio maps is time consuming, the radio maps will become outdated with any environmental change, different mobile devices read the received signal strength (RSS) differently, and peoples’ presence in LOS between access points and mobile device affects the RSS. This research proposes a new Adaptive Indoor Positioning System model (called DIPS) based on: a dynamic radio map generator, RSS certainty technique and peoples’ presence effect integration for dynamic and multi-floor environments. Dynamic in our context refers to the effects of people and device heterogeneity. DIPS can achieve 98% and 92% positioning accuracy for floor and room positioning, and it achieves 1.2 m for point positioning error. RSS certainty enhanced the positioning accuracy for floor and room for different mobile devices by 11% and 9%. Then by considering the peoples’ presence effect, the error is reduced by 0.2 m. In comparison with other works, DIPS achieves better positioning without extra devices.

## 1. Introduction

The location of a specific mobile device (MD) or user in a specific environment can be determined using positioning systems [1]. The Global Positioning System (GPS) is a satellite-based positioning system developed by the United States in 1973. The widespread usage of the GPS has shown the significance of Location Based Services (LBS) in people’s daily life [2]. LBS are defined as services that integrate a mobile user’s location and other data or information to provide more valuable information to that user [3]. LBS have been used in several areas and critical systems such as: military, healthcare, manufacturing, marketing, logistics and many other industries [4].

Despite its great use, the GPS service fails to accurately identify indoor locations due to the lack of line of sight (LOS) between GPS receivers and satellites. This limitation has pushed many researchers to explore other techniques and methods to enable LBS in such closed environments. Many technologies have been proposed to enable indoor LBS such as Radio Frequency Identification (RFID) [5], Infrared Radiation (IR) [6], Ultra-Wide Band (UWB) [7], Ultrasound [8], Bluetooth [9] and Wireless LAN (WLAN) [10,11]. An Indoor Positioning System (IPS) is a system that determines the physical location (floor, room, or point coordinates) of a MD within a closed environment [12].

Fingerprinting is a technique to identify MD locations based on RF features analysis. It operates in two different phases: the offline and online phase. The offline phase is considered the initialization phase, in which each site’s location in the desired environment will be surveyed to calibrate RF’s features, such as RSS values, of all the available Access Points (APs). Then the location coordinates and its respective calibrated RF’s features values will be stored to create the site’s fingerprint database which is called a radio map (RM). On the other hand, the current location of an MD, which is unknown, will be determined based on the online values of the calibrated RF’s features in the online phase. In the online phase, pattern recognition techniques or algorithms such as K-Nearest Neighbor (KNN) and Artificial Neural Networks (ANN) can be used as positioning algorithms [13]. 

Although fingerprinting is one of the most accurate positioning methods, the accuracy can decline due to many factors such as: occurrence of environmental changes, received signal strength (RSS) variation due to MDs’ heterogeneity [14], and the people presence effect (PPE) on RSS [15,16,17]. Such factors will affect the physical effects (reflection, refraction, diffraction, etc.) of the waves. Then initiating the RM database by surveying all the site locations to calibrate RSS is a time consuming process. 

Therefore, there is a real need for an adaptive indoor positioning model to provide accurate positioning results based on WLAN fingerprinting method for dynamic and multi-floor environments. Dynamic in our context refers to the people effect and device heterogeneity. This research proposes a new adaptive indoor positioning model based on path loss model to adapt WLAN fingerprints RM according to the occurrence of environmental changes. The proposed model is also able to overcome the MD heterogeneity problem and considers the presence of people as a WLAN signal’s attenuation factor.

## 2. Related Work

The related work in this section was organized in four different subsections. The first subsection explores the dynamic radio map updating solutions to overcome the environmental change occurrence. The second subsection provides some of the proposed solutions for multi-floor indoor positioning. The researches related to the effect of people presence are discussed in the third subsection. The final subsection presents and discusses some MD heterogeneity-related works.

### 2.1. Dynamic Radio Map

Bahl el al. [18] proposed RADAR as the pioneer of the IPS based on WLAN fingerprinting. It records and processes the WLAN’s RSS to determine the mobile user’s location. Then Krishnan et al. [19] provided Location Estimation Assisted by Stationary Emitters (LEASE) that uses a small number of stationary emitters and sniffers to avoid the time consuming RM calibration process. The experimental results show that LEASE can provide accurate localization in multi-floor environments, but it is considered a costly system since it needs stationary emitters and sniffers in each site in the environment. 

Xiaoyong et al. [20] showed empirically that reducing the sampling time or the number of location samples reduces the positioning accuracy rate. They then proposed Hidden Markov Model (HMM) and Expectation Maximization (EM) algorithms to reduce the RM calibration efforts without decreasing the accuracy. This method could not overcome the effects of the occurrence of changes because the manual RM calibration is still needed. Ji et al. [21] proposed a dynamic indoor localization method which uses a deterministic signal propagation model and ray tracing model to create a radio map and uses a Least Mean Square Error (LMSE) principle to determine the current position of a MD. The computational cost of the techniques used in the proposed solution decreases the IPS’ responsiveness. Another solution to overcome the time consuming map calibration process by creating an adaptive radio map based on inter-beacon co-calibration was proposed by Lo et al. [22]. In the training phase, two types of RSS need to be calibrated in the same time for each location in the site. They are Beacon-to-Device (BD_RSS) and Beacon-to-Beacon (BB_RSS). However, measuring the BB_RSS in real time is difficult to achieve. Then Narzullaev et al. [23] proposed an algorithm that combined the concept of the reference point (feedback point) and the one-slope-model (OSM) path loss signal prediction model to obtain a time-efficient calibration process. However, the algorithm increased the computational complexity by searching for the strongest APs and determining their locations. 

Chen et al. [24] proposed a sensor–assisted WiFi-indoor localization system to overcome the effects of the changes in the dynamic environment. The proposed system used three different technologies (RFID, Bluetooth, and HR sensors) in order to achieve the desired target. Using these technologies together means extra cost and computational burden for the environment infrastructure. Later Adaptive Localization with Enhancement (DALE) was proposed by Segou et al. [25] to overcome the effects of the occurrence of environmental changes without using complex hardware or feedback points. A number of beacons and APs were placed in each room in the desired environment. Then a simple algorithm was used to determine the location of a MD based on its measurements of the RSS of the available APs. The proposed algorithm needed at least four APs in each room which means it brought extra costs to the environment infrastructure. Chen et al. [26] proposed an adaptive WiFi positioning model that combined K-means algorithm and ANN in order to use a trilateration method to estimate the current position of a MD in a dynamic environment. This proposed model has high computional complexity since it used K-Means, ANN and included all the available MDs in order to determine one MD location. 

Atia et al. [27] proposed a system that dynamically and continuously calibrates a fine radio map by using the Bayesian regression algorithm to estimate the posterior RSS probability distribution over all locations based on online observations of the available APs and Gaussian prior centered over logarithmic path loss mean. It is difficult to update the APs’ firmware in order to collect RSS of the other APs and send it within its beacon frame and this will place a heavy load on the APs. Koweerawong et al. [28] created an adaptive RSS database by asking a feedback point to read the RSS of the available APs, then estimate the RSS for the remain locations of the site based on the nearest three feedback points, using feedback devices to cover 5% of the targeted environment site locations but this requires extra infrastructre.

Ali et al. [29] proposed a systematic localization approach, “LOCALI”, for indoor positioning. LOCALI generates RSS maps based on the environment plan, so it does not require a calibration database and extensive updates. LOCALI converts the environment plan into a pixel map of 10 pixel/m resolution. Then it generates a RSS map based on the pixel map and estimates the target location based on the generated RSS map. The experimental results show that LOCALI achieved accurate positioning result with 2 m of distance error. However, the proposed LOCALI did not consider the people presence effect and the mobile heterogeneity effect. In addition, it is not applicable in multi-floor environments.

### 2.2. Multi-Floor Indoor Positioning

Multi-floor positioning is considered as a challenge for indoor positioning. It is important to mention that multi-floor positioning-based WiFi-based fingerprinting for dynamic environments was listed as one a goals of the IPIN 2016 competition [30].

Liu et al. [31] provided IPS based on combining the fingerprinting method and path loss models for multi floor indoor positioning. A sample of reference points per floor were selected to create the fingerprints radio map and in the online phase the floor number was localized based on searching the radio map. After determining the floors, the MD location was determined by triangulation methods based on the APs’ location. A manual RM calibration will be needed in the case of any environmental change occurrence. Fan et al. [32] showed that the RSS is relatively sensitive to indoor obstructions, wall and ceilings, and on the other hand most of the existing localization algorithms are not suitable due to the complex indoor environment. In addition tof that the Multi-Wall Model (MWM) which was proposed in [33] only considered attenuation caused by wall number and texture and it did not consider the attenuation caused by the ceiling penetration in multi floor buildings. To overcome that problem the researchers proposed a sensor-based localization for multi-wall and multi-floor environments. It applied to wireless sensor networks (WSNs) and this means extra costs to the environment. 

Kejiong et al. [34] noticed that most of the IPS focused on relatively small environments. Then they proposed a location estimation system for a large multi-floor building using hybrid networks which are GSM and WLAN. The proposed solution was based on using compound RSS fingerprinting by measuring the RSS of all available APs’ and GSM signals. The searching of the most effective transmitter of the combined technologies, which are GSM and WLAN, increased the system computational complexity.

### 2.3. People Presence Effect

RADAR [18] researchers noticed that RSS in any location varied based on the orientation of the person who was calibrating it with respect to an AP. The researchers built a four orientations RM to overcome this problem. Unfortunately the proposed solution increased the calibration effort four times and it is not applicable for dynamic environments. Chen et al. [24] identified people presence as one of the main attenuation factors of WLAN signals and they assumed there are two different states, which are blocking and block-around, to reflect people presence near MDs. However, the block-around state is generally an infrequent state or case to represent peoples’ presence indoors. Hence, it needs more investigation about PPE on IPS positioning accuracy. 

Hamida et al. [35] observed the relationship between fluctuations in RSS and the presence of people activity within WLAN coverage areas. Unfortunately, the researchers did not mention the number of people in the environment during the daytime. Karadimas et al. [36] statistically proved that the signal strength can vary over time based on the human activity in the short-range 60 GHz wireless network. Turner et al. [37] studied the human movement in 2.4 GHz environments and proved experimentally that there is significant impact of the number of people and their movement speed on the signal strength, but this work considered only the case in which the human body obstructed the LOS and it did not provide any indication about RSS decline, in dBm, as the effect of a single human body. 

Fet et al. [38] mentioned that the manual RM building required orientation-dependent RSS calibration to overcome the signal attenuation caused by the human body. The researchers showed that WLAN signal distribution with distance takes an elliptical shape due to the presence of people. Then, based on the properties of the ellipse and some empirical measurements they proposed a RSS distribution model to generate a multiple orientation RM depending on the 0° direction calibration. However, using multi-orientations RM means extra computational burden for the whole IPS.

People presence was listed as signal attenuation factor in many studies, but more investigation is needed to overcome this effect in IPS. Bahl et al. [18] and Fet et al. [38] used the concept of multi-orientation RM to overcome the effect of people presence on IPS positioning results which either requires a huge human effort or does not fit MDs’ limitations. On the other hand, Chen et al. [24] used an infrequent case by considering the block-around state and the rest of the discussed works either studied the effect of PPE on RSS. However, PPE on RSS need to be modeled and integrated to gain accurate positioning result. 

### 2.4. MD Heterogeneity

The proposed solutions for the MD heterogeneity problem can be categorized as manual solutions [39] or automatic solutions [40]. The subsequent solutions, named calibration-free solutions, aim to find suitable RSS transformations by extracting common features from RSS tuples in the online phase without any extra calibration [41,42].

Hossain et al. [43] proposed to use the Signal Strength Difference (SSD) values between each pairs of the received signal to replace the absolute signal strength value as a location fingerprint. It can be considered as a costly solution because SDD relied on the combination $\left(\begin{array}{c} n\\ 2 \end{array}\right)$ of extracted features where n is the number of the available APs. Kjaergaard and Munk [44] proposed the Hyperbolic Location Fingerprinting (HLF) method that replaced the absolute signal strength with the ratio between each pair of received signals. However, the accuracy of such an approach is still low (52%) so MD heterogeneity still needs a better solution. 

The usage of Spearman Ranking Correlation coefficients was proposed by Jimenez and Ruizhong [45] to find the correlation between RSS tuples’ items. However, the achieved positioning accuracy only reached 50% which is low and its computational cost is high, which decreases the responsiveness features of the positioning system. Luo and Zhan [46] suggested to adjust the RSS Gaussian curve fitting by considering the skewness and kurtosis coefficients to ensure accurate modelling of RSS, but this needs extra statistical processing which will lead to a high computational cost. Lyu-Han [42] proposed a calibration-free indoor positioning algorithm based on two common features of different MDs which are RSS order and the linear dependency between the measured RSS by different MDs. Their experimental work showed that the proposed algorithm can achieve a small distance error (1.8 m). Although the proposed algorithm achieved acceptable accurate positioning results, it is considered as a costly solution in terms of responsiveness since it uses complex computation. 

The previous solutions can be considered good solutions to handle the MD heterogeneity problem. However, none of these can be considered as mature solution to overcome this problem since the achieved positioning accuracy still low and requires high computational loads. This means that more investigation is needed to overcome the problem. 

## 3. Methodology

**Figure 1:**
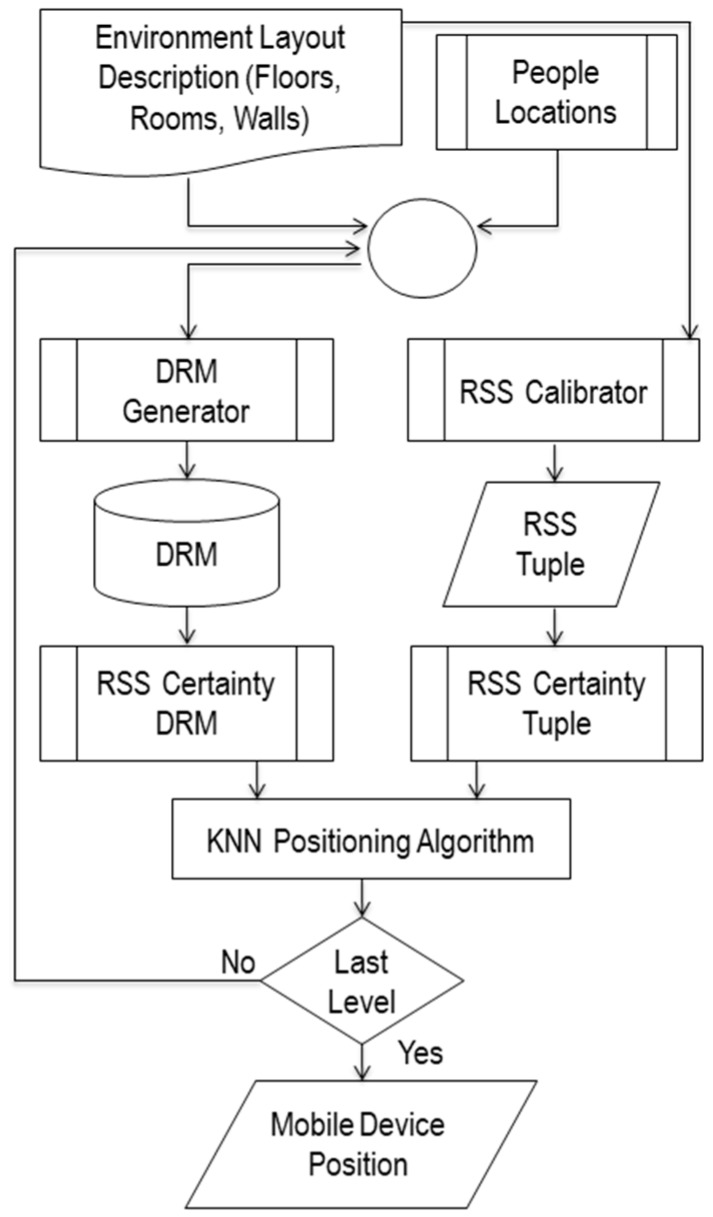
The proposed adaptive indoor positioning model (DIPS).

The model of the proposed system is illustrated in Figure 1. It combines and extends our previous works described in [47,48,49,50,51]. 

It starts when the current location of MD is required. Then, the RSS Calibrator calibrates the RSS-tuples of the available APs, where the available APs list can be extracted from an Environment Layout Description (ELD). In parallel, the Dynamic Radio Map (DRM) Generator will generate DRMs for the desired positioning level based on ELD and People Locations. Then both of the DRM and RSS-tuple will be passed to RSC component to convert it to RSC-DRM and RSC-tuple. Finally, the positioning algorithm will estimate the current positioning of MD, as a 3-tuple positioning result (floor, room, point), based on the RSC-DRM and RSC-tuple. The decision is used to control the hierarchal DRM generation for the different positioning levels floor, room and point. 

The ELD component contains information about environment such as the area, the existing floors and the available rooms, walls and APs in each floor. All this information is required to generate a DRM by using the modified path loss model in Equation (1). $\mathrm{PL}(d_{0})$ is the free space propagation loss at reference distance $d_{0}$ (typically 1 m), n is the slope factor (power decay index) which becomes 2 for free space and 6.5 for obstructed space [52]. ∑WAF, ∑FAF, and ∑PAF are the sum of the attenuation factor of all the walls, floors, and people between AP and reference point. X is a zero-mean Gaussian distributed random variable:

(1) $$\mathrm{PL}\left(d\right)=\mathrm{PL}(d_{0})+10n\log\left(\frac{d}{d_{0}}\right)+\sum^{\text{​}}\mathrm{WAF}+\sum^{\text{​}}\mathrm{FAF}+\sum^{\text{​}}\mathrm{PAF}+X$$

People’s location component includes information about the existing people locations which are identified by floor number and position coordinates. This information will be used to consider the effect of people presence in the LOS between AP and MD as a signal attenuation factor to integrate it into DRM generator as seen in Equation (1). The DRM generator (DRMG) is a key component that will be used to generate RM fingerprints based on the path loss model shown in Equation (1). It generates DRM upon the incoming positioning request, and generates three DRMs for floor, room and point positioning levels, respectively. First, Floor DRM (FDRM) will be generated based on the APs and floor information only. APs information consists of RSSI and MAC address. The algorithm is shown in Algorithm 1. FDRM consists of a small number of reference points (maximum eight reference points per floor), each of which is labeled with the floor label (FL). We just use a small number of reference points to make a light RM which can fit with the MDs’ limitations. The algorithm starts by dividing the area of each floor into a number of sub-areas (two, four, and eight sub-areas) which each of them has same area as explained in line 1 to line 8. The center point of each sub-area of each floor will be used as reference point in this FDRM. Then the DRMG will use these reference points to form the target FDRM.

**Algorithm 1:** Floor Dynamic Radio Map (FDRM) Generation
**Input**: Area W&L, Floors List, AccessPoints Lis
**Output**: Floor Dynamic Radio Map (FDRM)**1:**Create Reference Point List (RP)**2:**For i ← 1 to 4 do**3:**  For j ← 1 to 2 do**4:**  rp.x ← (i*2−1)*L/8**5:**  rp.y ← (j*2−1)*W/4**6:**  RP.add(rp)**7:**  End For**8:**End For**9:**Create Floor Dynamic Radio Map List (FDRM)**10:**For each FN in Floors List do**11:**  For each RP in Reference Point List do **12:**  For each AP in AccessPoints List do**13:**  d ← EuclideanDistance(RP, AP)**14:**  fb ← Absolute(FN − AP.floor)**15:**  rss ← −34−22*log10(d)−fb*26 − random(−1,1)**16:**  FDRM.add(rss+FN)**17:**  End For**18:**  End For**19:**End For**20:**Return FDRM

RSS Calibrator, represents the MD, initiates the positioning process by calibrating RSS from the available of APs online. RSS Calibrator gets the available APs list from the ELD, so it can filter collected beacon frames based on this list. Received Signal Strength Certainty (RSC) is the component that is responsible for addressing the MD heterogeneity problem. RSC component converts the generated DRM instances (RSS tuples) and then the MD calibrated RSS tuple into RSC tuples in order to determine the current location of MD. 

Then RSC, DRM and RSC tuple will be passed to the positioning algorithm to find the current location of an MD. The positioning algorithm is the component that will be used to determine the current location of the MD based on the RSC RM and RSC tuple. KNN and ANN were nominated to be used as positioning algorithms.

### 3.1. Dynamic Radio Map Generation (DRGM)

DRMG is a solution to overcome the manual RM calibration issue in multi-floor environments and to overcome the effect of any environmental change occurrence on IPS. First, manual RM was calibrated over the selected testbed site. Second, an experiment was conducted to adopt one positioning algorithm from the nominated positioning algorithms to be used in the next step. Third, dynamic RM (DRM) was generated based on path loss model for multi-floor indoor positioning. The generated DRM was validated in three positioning levels which are floor positioning, room positioning and point positioning. Furthermore, for each positioning level a specific DRM was generated. The positioning process started by generating Floor-DRM (FDRM) for floor positioning as described in Algorithm 1. Then based on the floor positioning result, room positioning was performed by generating Room-DRM (RDRM) for the selected floor. Finally based on the room positioning result, the point positioning level was performed by generating Point-DRM (PDRM) for the selected room.

#### 3.1.1. Manual Radio Map Calibration

**Figure 2:**
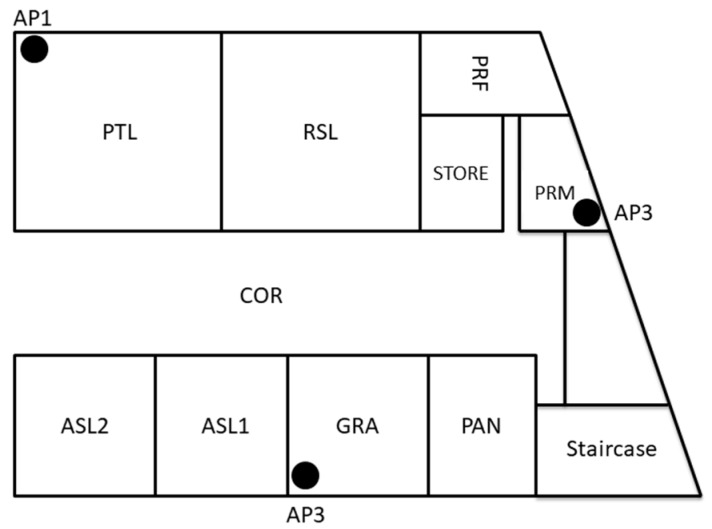
The selected testbed diagram—Menara Razak Level 3.

The eastern side of the 3rd floor in Menara Razak (Figure 2) was gridded into small areas of size 1 m^2^, and each cell represented a reference point in the targeted manual RM. The area of the selected site is 300 m^2^ which is covering 300 different reference points for RSS calibrations. We collected 30 beacons at each reference point. Then, RSS values of the three APs as shown in Figure 2 were extracted from the beacon dataset to create the first manual RM. This manual RM was labeled as the Raw Radio Map (RRM). Then to overcome the possible effect of RSS fluctuation on the positioning result, each reference point in the site was represented by the average value of the calibrated RSS of each AP which named as Averaged RM (ARM) and it has only 300 instances. ARM can be considered as a normalized calibrated RM.

#### 3.1.2. Positioning Algorithm Adoption

The nominated positioning algorithms (KNN and ANN) were trained and tested two times by using 10-fold (10 splits) cross-validation process based on the calibrated RMs. First, they were trained and tested based on RRM and their accuracy were determined using 10-fold cross-validation process; Second, they were trained and tested based on ARM and their accuracy were determined using 10-fold cross-validation process; Finally, the achieved results were compared, and the positioning algorithm with the highest positioning accuracy was adopted. 

#### 3.1.3. DRM Generation for Multi-Floor Environments

The initial step is to determine the parameters of the path loss model. Then the next step is DRM generation for multi-floor environments. The results are the FDRM, RDRM and PDRM, which are the floor, room and point positioning levels, respectively. The accurate estimation of a specific AP’s RSS from a certain reference distance depends on the accurate values of the parameters of the path loss model. Determining these parameters accurately requires a good understanding of the targeted indoor environment. The targeted parameters are path loss at a reference distance, path loss exponent, wall attenuation factor (WAF) and floor attenuation factor (FAF).

To define the path loss at a reference distance, three APs and MD were fixed at the same high with a reference distance d_0_ that is equal to 1 m. APs were configured to generate four beacon frames per second, and the MD was configured to scan the air for the available APs beacon frames four times per second for 10 min. The duplicated beacon frames were deleted to remove any bias. This duplication occurred because of the difficulty to synchronize time between the access points and the mobile devices. Then in order to estimate the path loss exponent in the selected environment, RSS of the available three APs were calibrated with over 30 reference points in ASL2, ASL1, GRA rooms, and the corridor in front of it as shown in Figure 2. Then based on the coordinates of both the APs and the reference points, the OSM path loss propagation model was used to estimate the path loss exponent value for each access point. Then the mean of path loss exponent values for all APs was computed.

The layout of the selected environment has been determined by plaster walls with a thickness of 5 cm each. To estimate WAF in the selected environment, AP and MD were fixed with a distance of 2.05 m in between. In the first case, AP and MD were in LOS, while in the second case AP and MD were rotated 90° to have NLOS between AP and MD with a wall in the middle of the distance. FAF, which is caused by the ceilings in multi-story environments, can have a high impact on RSS. According to the literature, the ceiling’s thickness and material determine its attenuation factor. To estimate FAF value in the selected environment, RSS was calibrated for more than 5 min in the two cases. First, AP and MD were fixed on a vertical line where the AP was fixed on the fourth floor tile and MD was fixed on the third floor tile; Second, AP and MD were fixed on a horizontal line with a distance of 4 m in between, which is the floor’s overall height. 

DRM was generated automatically to overcome the effect of the environmental change occurrence for multi-floor indoor positioning. The generated DRM size will vary based on the targeted positioning level. First, a light FDRM was generated with a small number of reference points; Second, RDRM was generated based on the output of the floor positioning level. The center point of each room in the selected floor represents one reference point in RDRM; finally, PDRM was generated based on the output of the room positioning level. The selected room was gridded into cells with an area of 1 m^2^ and each cell presents a reference point in PDRM. A multi-floor RM contains a huge number of reference points, and this makes it unsuitable due to the limitations of the MD. To overcome this, a light FDRM was generated to meet MD specification for floor positioning. After the validation process, only one of these FDRMs was adopted for floor positioning level. 

The generated DRMs were used as the basis for WLAN fingerprinting-based IPS. KNN was used as a positioning algorithm. Experiment started by calibrating RSS at fifty reference points over three different floors in the selected testbed which are Level 3, Level 4 and Level 5 in Menara Razak building. Most of these reference points were chosen in the main corridors and some different rooms or offices in the different floors. Two validation processes were conducted, the first process was conducted with KNN (k = 1) and the second one with KNN (k = 3). 

### 3.2. Received Signal Strength Certainty (RSC)

We propose RSC because it can be a robust solution for the MD heterogeneity problem. There are three phases in RSC. The first phase aims to show the effect of MD heterogeneity on RSS. Hence, different MDs will be used to calibrate RSS simultaneously at different reference points. Then the calibrated RSS will be presented to show the effect of MD heterogeneity on the calibrated RSS and to extract patterns from these RSS values to address the MD heterogeneity problem. The second phase aims to present and validate RSC as a robust solution for the MD heterogeneity problem. This validation includes a mathematical proof for RSC concept. The third phase aims to validate RSC effect on indoor positioning result to show the efficiency of RSC as a robust solution for the MD heterogeneity problem. This validation includes its effect on the three levels of positioning floor, room and point positioning. In addition, a comparison between RSC and some of the most recent related works will be conducted. RSC must be applied on the generated DRM as well as the online calibrated RSS tuple, then RSC component appear twice in the proposed DIPS. 

#### 3.2.1. MD Heterogeneity Effect of RSS

Three different MDs (ASUS Zenphone4, LENOVO A386, and Samsung TAB4) were used to calibrate the RSS of the available APs simultaneously at different reference points. These reference points were located on different floors, and different rooms on the eastern side of Menara Razak (Level 4 and Level 3). Then the calibrated RSS, 150 beacons for 5 min over each point, were presented in line chart to explore and extract patterns from these RSS values. This extracted pattern was used to address the MD heterogeneity problem. 

In order to show the MD heterogeneity effect on different floors, MDs were calibrated RSS of the available APs in Level 3 (AP31 and AP32) and Level 4 (AP41 and AP42) at two different points. These two points were chosen in the center of Level 3 and Level 4, respectively. 

#### 3.2.2. Theoretical RSS Presentation

To understand the concept behind the RSC, assume that there are n AP in the selected environment and assume that MD represented RSS of the all AP as n-tuple $\left({\mathrm{rss}}_{1};{\mathrm{rss}}_{2};\;\ldots \;;{\mathrm{rss}}_{n}\right)$ where ${\mathrm{rss}}_{i}$ is RSS of the i*th* AP. Now, RSC tuple is $\left({\mathrm{rsc}}_{1};{\mathrm{rsc}}_{2};\;\ldots \;;{\mathrm{rsc}}_{n}\right)$ where ${\mathrm{rsc}}_{i}=\frac{{\mathrm{rss}}_{i}}{\sum_{1}^{n}{\mathrm{rss}}_{j}}$.

In order to prove that the different RSS tuples—which were calibrated at the same reference point and time by different MDs—have similar RSC tuples mathematically, one-to-one and onto function must be found between different RSC tuples. Therefore, a proof for the following hypothesis must be provided. The hypothesis is “For each different RSS tuple $T_{1}\;\mathrm{and}\;T_{2}$, which were calibrated by MD1 and MD2 at the same reference point simultaneously, there are two similar RSC tuples ${T_{1}}^{\prime }\;\mathrm{and}\;{T_{2}}^{\prime }$ where ${T_{1}}^{\prime }=\mathrm{RSC}\left(T_{1}\right)$ and ${T_{2}}^{\prime }=\mathrm{RSC}\left(T_{2}\right)$”. 

In order to prove this hypothesis, assume that $T_{1}\;\mathrm{and}\;T_{2}$ are two different RSS tuples for MD1 and MD2 respectively, and ${T_{1}}^{\prime }\;\mathrm{and}\;{T_{2}}^{\prime }$ are the corresponding RSC tuples of $T_{1}\;\mathrm{and}\;T_{2}$ respectively. So that based on Equation (2) we need to show that the i*-th* items $p_{i}$ and $q_{i}$ in each RSC tuple, ${T_{1}}^{\prime }\;\mathrm{and}\;{T_{2}}^{\prime }$, are similar with different constants {a, b} and {x, y} respectively where $p_{i}=a\times S_{i}+b$ and $q_{i}=x\times S_{i}+y$. Hence, the proof will start by Equation (3):

(2) $$Pm_{i}=a_{i}\times P\left(d\right)+b_{i}$$

(3) $$\frac{p_{i}}{\sum p_{i}}\;≅\;\frac{q_{i}}{\sum q_{i}}$$

then by replacing $p_{i}$ and $q_{i}$ in the previous Equation (4) then it becomes as Equation (4):

(4) $$\frac{a\times s_{i}+b}{a\times \sum s_{i}+\mathrm{nb}}\;≅\;\frac{x\times s_{i}+y}{x\times \sum s_{i}+\mathrm{ny}}.$$

Let us define function T: R→R as in Equation (5):

(5) $$T\left(x\right)=T\left(\left(\frac{a}{\sum^{\text{​}}p_{i}},\;\frac{b}{\sum^{\text{​}}p_{i}}\right)\left(\begin{array}{c} r\\ t \end{array}\right)\right)$$

where r and t any two real numbers, then we can define y as in Equation (6):

(6) $$\to \left(\frac{x}{\sum^{\text{​}}q_{i}},\;\frac{y}{\sum^{\text{​}}q_{i}}\right)\left(\begin{array}{c} r\\ t \end{array}\right)=y$$

This value is valid since ∀ x∈R r and t can be taken as $\frac{x}{2a}\sum^{\text{​}}p_{i}$ and $\frac{x}{2b}\sum^{\text{​}}p_{i}$ respectively, and T is a linear transformation as in Equation (7):

(7) T(γx+y)=γT(x)+T(y)

hence:

(8) $$T\left(\frac{p_{i}}{\sum^{\text{​}}p_{i}}\right)=T\left(\left(\frac{a}{\sum^{\text{​}}p_{i}},\;\frac{b}{\sum^{\text{​}}p_{i}}\right)\left(\begin{array}{c} s_{i}\\ 1 \end{array}\right)\right)$$

(9) $$=\left(\frac{x}{\sum^{\text{​}}q_{i}},\;\frac{y}{\sum^{\text{​}}q_{i}}\right)\left(\begin{array}{c} s_{i}\\ 1 \end{array}\right)=\;\frac{{\mathrm{xs}}_{i}+y}{\sum^{\text{​}}q_{i}}=\;\frac{q_{i}}{\sum^{\text{​}}q_{i}}$$

(10) $${T_{1}}^{\prime }\approx {T_{2}}^{\prime }$$

This means ${T_{1}}^{\prime }\;\mathrm{and}\;{T_{2}}^{\prime }$ are similar and they have the same properties.

#### 3.2.3. Practical Validation of the RSC Effect

This section provides practical validation of the effect RSC on the indoor positioning accuracy in different positioning levels: floor, room and point positioning levels. For this task, the DRM generation was adopted to be the base for any positioning process. In addition, two different testing sets were calibrated by using three different MDs: ASUS; LENOVO and TAB4. The first testing set, which was named as Floor Heterogeneous MD Testing set (FHMDT), was used to validate the effect of RSC on floor positioning and the second testing set, which was named as Heterogeneous MD Testing set (HMDT), was used to validate the effect of RSC on room and point positioning levels.

8FDRM was generated for floor positioning in this validation level. RSC was applied on both of 8FDRM and FHMDT. In addition, KNN algorithm with k = 1 was used as positioning algorithm. This adoption was carried because 8FDRM and KNN with k = 1 achieved the best positioning result for floor positioning level. 

### 3.3. People Presence Effect

According to the literature, peoples’ presence under the WLAN coverage affects its signal strength. Although this effect may lead to inaccurate positioning it is hard to find research that involved peoples’ presence in their proposed WLAN-based indoor positioning due to the unpredictable behavior of people. In this section, the effect of the presence of people between AP and MD was investigated experimentally. This investigation aimed to model PPE on RSS in order to integrate PPE into DRG. To achieve this, untechnical information such as people allocation or queueing psychology in closed environments is needed. This information will be used to make assumptions for formulating PPE as attenuation factor. Then this formulation will be included to the proposed IPS model. 

#### 3.3.1. People Allocation Psychology

There is no rule that controls people allocation psychology indoor due to their unexpected behavior. Proxemics is a famous psychological concept which talks about the spatial requirements of humans and the effects of population density on their behavior, communication, and social interaction [53]. Proxemics theories stated that people always keep at a distance from others in order to have a special space or zone to find their privacy. This space varies according to the relation between the person and the surrounding people [54]. This means that people are always allocated irregularly in order to keep at a distance from other people. Edward Hall, an American anthropologist, presented the concept of reaction bubbles. The reaction bubbles theory states that there are four different zones surrounding any person which are intimate space, personal space, social space and public space. Intimate space is the closest space which is used for embracing, touching or whispering and its distance is less than 0.46 m. The personal space distance ranges from 0.46 cm to 1.2 m and it used for interactions between close friends or family. The social space used for interactions with colleagues or associates and it distances ranges from 1.2 m to 3.7 m. Finally, the public space ranges from 3.7 m to 7.6 m or more and it always used for public speaking or talking to a large group.

In this research, 1 m of distance will be adopted as personal space for people who involved in the subsequent experimental work. This adoption fitted with the environment gridding and it maintains a social zone between people in the environment. This social zone is an intermediate zone between personal space and public space as shown in personal reaction bubbles figure. 

#### 3.3.2. PPE in Horizontal LOS

In order to show PPE in HLOS, this section provides the design of three different experiments. These experiments have the same configurations but they differ in their people presence scenarios. Furthermore, in this experiment both of the used AP (CISCO WAP4410N Wireless-N, Cisco, Taipei, Taiwan) and MD (Samsung Tab 4-T231, Samsung Electronic, Binh Duong, Vietnam) were fixed on 0.75 m height portable cabinets with 3 m of distance between them. AP was configured to generate four beacons frames per each second and MD was configured to calibrate RSS, of 200 beacons over seven minutes, per scenario.

#### 3.3.3. PPE in Diagonal LOS

Wireless experts advise placing APs in high places, such as ceilings, in order to have the best coverage. Hence, diagonal LOS (DLOS) between AP and MD is the most common case in the indoor environments. In order to show PPE in DLOS, this section provides the design of two different experiments. The first experiment aims to show PPE on RSS in corridor with 2.5 m of width. The second experiment aims to show PPE on RSS in free space. Then the calibrated RSS will be analyzed in order to have general view bout PPE on RSS and its distribution in the case of DLOS. In the first experiment, RSS was calibrated in a corridor, with 2.5 m of width and different scenarios of people presence. The RSS centroid values and its distribution values were extracted from the descriptive statistics of the calibrated RSS. 

#### 3.3.4. PPE in Virtual LOS

**Figure 3:**
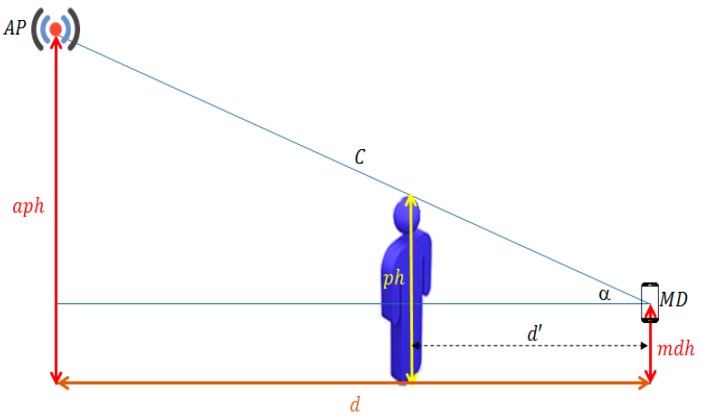
PPE influence distance ($d^{\prime }$) based on triangle geometry.

**Figure 4:**
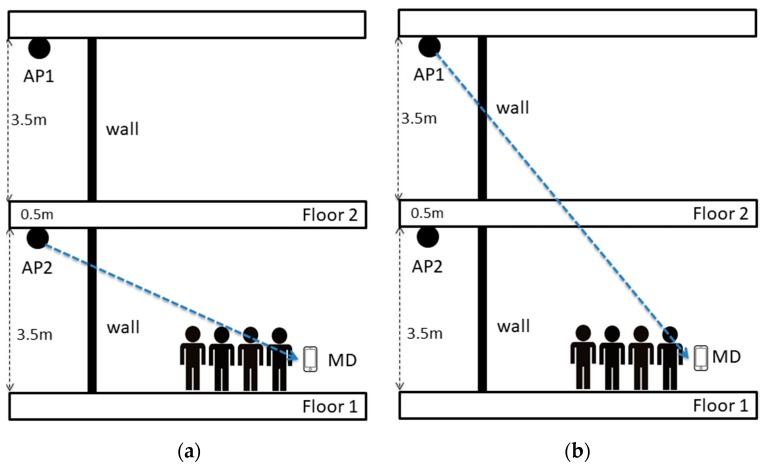
PPE in VLOS between AP and MD. (**a**) VLOS case1—one obstacle cut LOS; (**b**) VLOS case2–two obstacle cut LOS.

PPE in Virtual LOS (VLOS) means people presence in the virtual line on LOS in the case on NLOS as shown in Figure 3. In order to show PPE in DLOS, this section provides the design of two different experiments. The first experiment aims to show PPE on RSS in VLOS where a wall obstacle LOS between AP and MD as shown in Figure 4a. The second experiment aims to show PPE on RSS in VLOS where a floor and a wall obstacle LOS between AP and MD as shown in Figure 4b.

#### 3.3.5. Defining PPE Influence Distance (PPID)

Path loss models show a negative correlation between RSS and the distance between AP and MD. In this phase, a new concept related to this correlation will be presented and it is named as PPE Influence Distance (PPID). PPID can be defined as the distance in which peoples’ presence can hinder the LOS line between AP and MD. Although peoples’ presence between AP and MD affects the RSS, this effect varies according to distance between the people and the MD. This concept was developed based on the assumption that the AP position is higher than the MD position as a result of the best practice to locate AP indoors. Hence, the influence distance must be determined based on the location of both AP and MD. Figure 3 presents a general geometry view of AP and MD in indoor environments. Here aph is the AP height from the horizontal line with MD, d is base distance between AP and MD in horizontal line, C is hypotenuse C “LOS length between AP & MD”, α is Alpha Angle, ph is person height 1.5–1.7 m, mdh is mobile device height and $d^{\prime }$ is person height influence distance. Based on the triangle geometry, the influence distance $d^{\prime }$ was formulated as in Equation (11):

(11) $$d^{\prime }=\frac{ph-mdh}{tan\left(\alpha \right)}$$

and since:

(12) $$\tan\left(\alpha \right)=\frac{aph-mdh}{d}$$

then:

(13) $$d^{\prime }=\frac{d\times \left(ph-mdh\right)}{\left(aph-mdh\right)}$$

This means that the influence distance concept is theoretically true, as shown in the previous Equations (11)–(13), and we need to show its validity practically. In order to show the validity of the influence distance, many experiments were conducted in different places with different numbers of people. The aim of these experiments is to show that PPE on RSS will vanish when the person’s presence could hinder the LOS between the AP and MD. The experimental work for the DLOS case was designed with the following measurements: *aph* = 3.5 m, *mdh* = 0.8 m, *ph* = 1.6 m and *d* = 8.8 m. The influence distance in this case is 2.6 m. Then the experimental work for the VLOS case 1 was designed with *aph* = 3.5 m, *mdh* = 0.8 m, *ph* = 1.6 m and *d* = 9.6 m. Hence, the influence distance in this case is 2.9 m. Finally, *aph* = 8 m, *mdh* = 0.8 m, *ph* = 1.6 m and *d* = 9.6 m was used in the experimental work for VLOS case 2 and the influence distance in this case is equal to 1.3 m.

#### 3.3.6. Integrating PPE into DRMG

**Figure 5:**
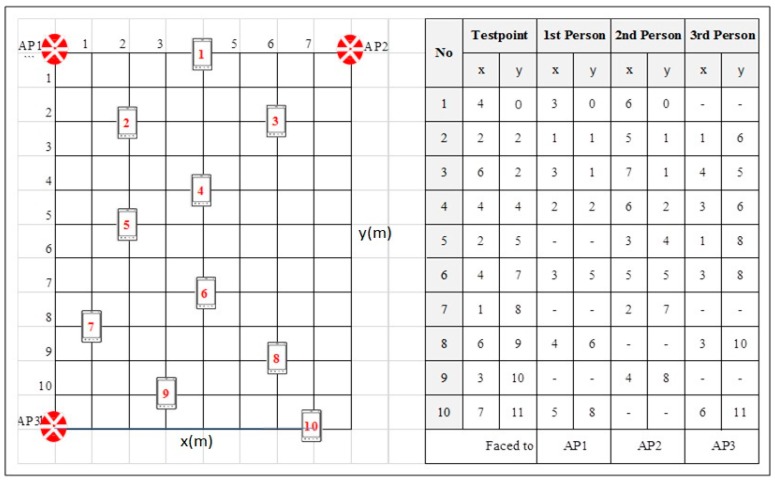
People allocation for each testing point.

In this section PPE was considered in the PDRM generation process. Hence, PPE within the influence distance was added to in the proposed path loss model as People Attenuation Factor (PAF) to generated PDRM for point positioning as shown in Equation (1). Then the effect of integrating PPE as PAF was validated in free space area. The RSS of the used APs was calibrated in ten different points with people presence in the LOS between MD and APs. Figure 5 shows the coordinates on these testing points and listed the coordinates on which people were located in LOS between MD and APs. Distance error was used as performance to validate the effect including PPE in PDRM generating. 

In order to validate PPE on IPS positioning results, an experiment was conducted in 88 m^2^ of free space area and RSS of APs was calibrated at ten different points with people presence in the LOS between APs and MD as shown in Figure 5. The all of people presence case occurred at a distance less than the computed PPID. This is because PPE on RSS must occur to validate the effect of considering PPE, in PDRM, on point positioning results. PDRM was generated for the grid area based on the path loss model in Equation (1) excluding WAF and FAF since the selected testbed is free space area. Two PDRMs were generated the first one, named is PDRMT, did consider PPE and the second one, named as PDRMP, considered PPE. Hence, KNN was used to find the coordinates of the current location of MD for each testing points against PDRMT and PDRMP as well. 

## 4. Results and Discussion

### 4.1. Performance of Dynamic Radio Map Generation

A manual dataset of beacon frames collected over 300 reference points in two different sessions was created based on the experiment explained in Section 3.1.1. Each session took more than 10 h of work to obtain a dataset with 9000 instances, in which 30 beacon signals were collected for each 300 reference points. This means that, by the end of the second session, a beacon frame dataset with 18,000 instances was created after 20 h of work. After removing the duplicate instances which occurred due to unsynchronized time between APs and MD, there were 4900 unique instances in the collected dataset. The experiment was conducted using three APs for each floor.

**Figure 6:**
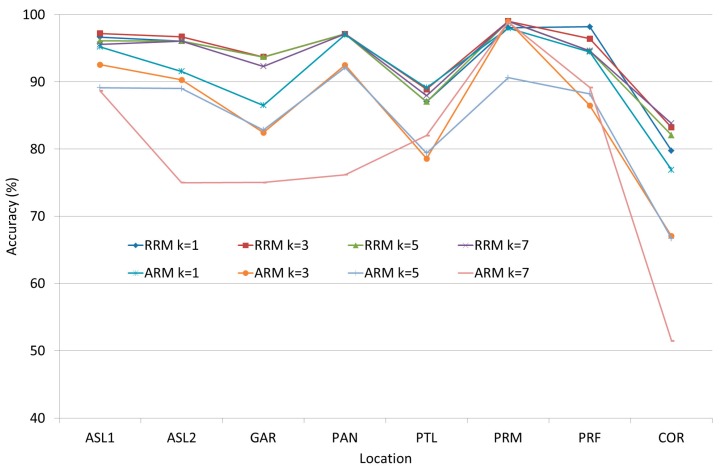
The accuracy of IPS when using KNN in each room.

**Figure 7:**
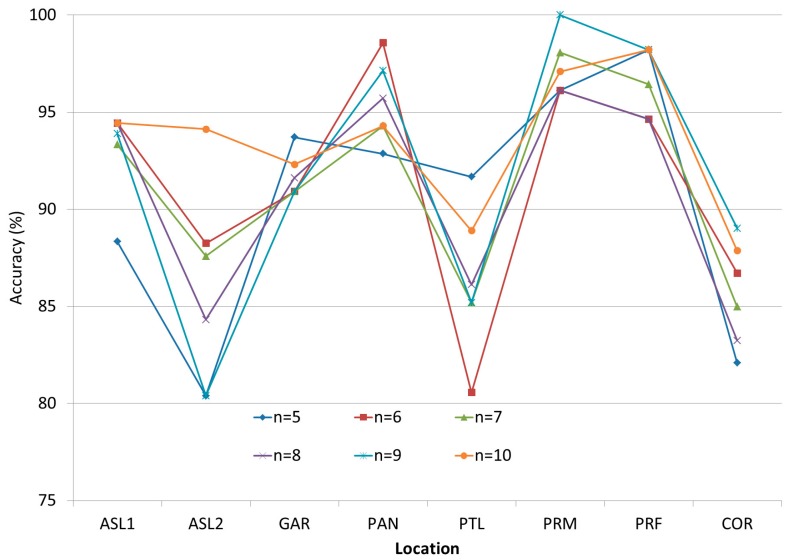
The accuracy of IPS when using ANN in each room.

The detailed results of the KNN and ANN experiments explained in Section 3.1.2 can be seen in Figure 6 and Figure 7. These testing points were located in different rooms or locations such as academic staff lounge 1 (ASL1), academic staff lounge 2 (ASL2), graduate assistant room (GAR), pantry (PAN), professional training lab (PTL), research lab (RSL), prayer room male (PRM), prayer room female (PRF) and corridor (COR). The lowest obtained positioning rate, which is 79.77% (RRM) or 51.47% (ARM), was in the corridor because the tight width of the corridor increases the signal reflection. This reflection increases the fluctuation of RSS. Most of the confidence intervals range between ±1 to ±3. 

**Figure 8:**
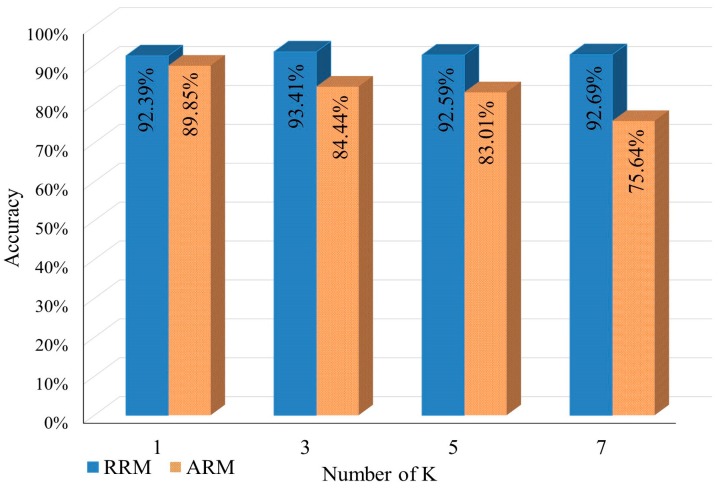
KNN positioning result accuracy.

**Figure 9:**
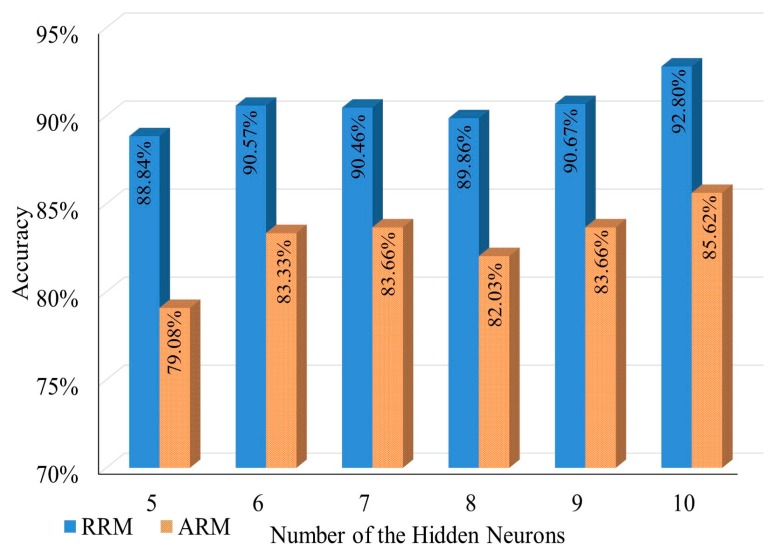
ANN positioning result accuracy.

The average accuracy of IPS when using KNN and ANN can be seen in Figure 8 and Figure 9. The obtained results showed that KNN could achieve more accurate positioning results than ANN. On the other hand, ANN had two weakness other than the poor performance. First, ANN is a model-based machine learning algorithm, which means that the system should retrain the network in case of environmental change occurrence; Second, ANN is a complex system that needs heavy computations especially in the training phase, and this complexity does not fit with mobile device limitation. Hence, KNN algorithm was adopted to be the fingerprint matching algorithm based on its acceptable accuracy and its relevance to the limitations of mobile devices.

Based on the experiment described in Section 3.1.3, path loss at a reference distance is −34 dBm. Path loss exponent (n) or power decay index ranges from 1.6 to 2.9 in the selected environment. The linear equations of the computed path loss exponent indicate that the computed path loss exponent values for the three APs are too close to constant coefficients because the coefficients on the x variable are too small and it can be neglected. This means that there are three different path loss exponents based on the different three APs and RSS calibration points in the same environment. The path loss exponent of this environment has been set to 2.2. 

**Figure 10:**
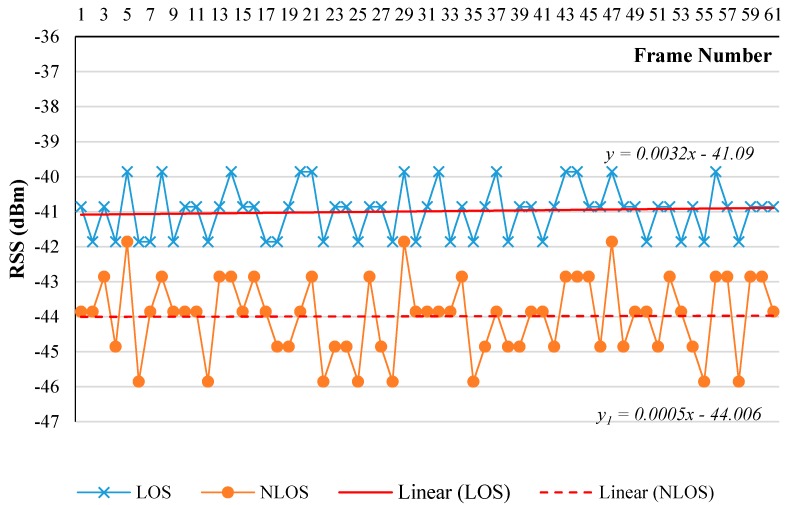
Wall attenuation factor (WAF) estimation.

Furthermore, the difference between the constant coefficient of NLOS (44.006) and the constant coefficient of LOS (41.090) represent the existing wall attenuation factor as shown in Figure 10. Hence, −3 dBm, the difference between the two constant coefficients was adopted as WAF in this research.

**Figure 11:**
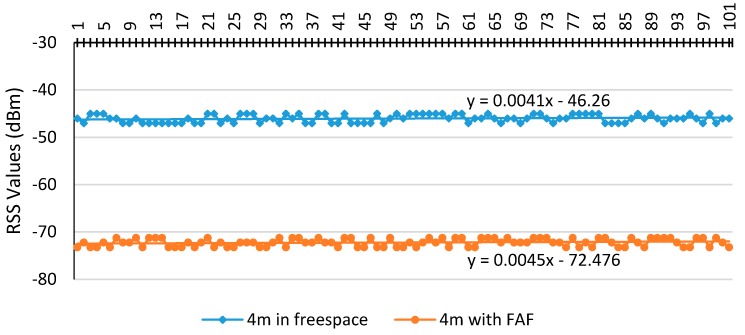
Floor attenuation factor (FAF) estimation.

Figure 11 shows the calibrated RSS on both of the LOS and NLOS cases. The described cases show that there is −26.21 dBm difference between their constant coefficients. This difference represents FAF of the existing ceiling. Hence, in this research −26 dBm was adopted as the FAF. 

**Figure 12:**
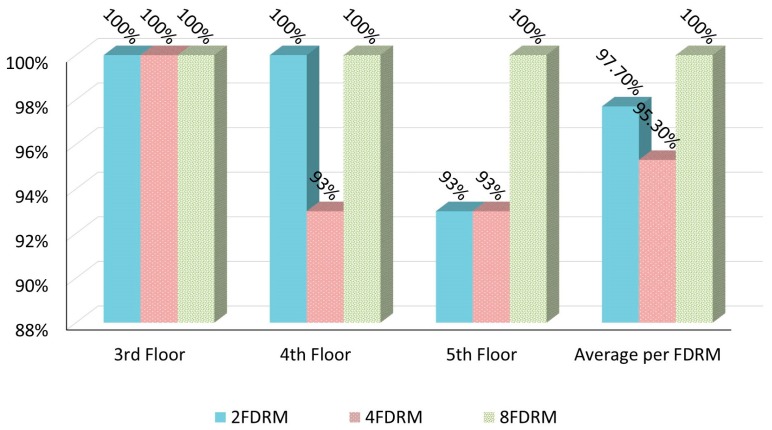
Floor positioning accuracy rate with k = 1.

Figure 12 shows the result of the first validation process. The overall results showed that the proposed model can achieve highly accurate floor determination in the indoor positioning process with a positioning accuracy that exceeds 98% on average. Of the obtained results, the highest accuracy rate (100%) occurred when k (in kNN) was set to 1 and the reference points were set to 8 (8FDRM) in each floor. This high accuracy occurred due to two factors: (1) the accurate determination of the path loss model parameters which lead to generate accurate represented radio map for each floor; (2) The high attenuation of RSSI due to floor attenuation factor which lead to high differences in RSSI between different floors.

The higher rate occurred with k = 1 because the similarities between reference points located in different floors is lower than the similarities between reference points in the same floor due to FAF. On the other hand, the false positioning results occurred only within the elevators and stairs area. This result was expected because these areas are considered a challenge for any wireless communication due to their unique characteristics such as the thickness of the concrete walls, huge amounts of metal, and the movement of elevators. 

After determining the floor number, the layout description of the selected floor was used to generate RDRM for the floor. The center point of each room was used as a reference point in RDRM. Each reference point was labeled with the room label based on the environment description. The existing walls were considered in RDRM generation with WAF = −3 dBm. In order to validate the proposed DIPS, the generated RDRM for room positioning level, two different testing sets named tRRM and tARM were extracted from the manual RRM and ARM respectively. The size of tRRM and tARM testing sets are 986 and 38 instances respectively and it represented 38 testing points were selected randomly from the manual calibrated RM. KNN with k = 1 was used to retrieve the room label for each instance in the previously described testing sets. The achieved positioning accuracy exceeded 92%. Point positioning is the last step. The efficiency of the proposed method in point positioning was validated by the distance error performance metric.

After determining the room in which MD was located, only the points within the predetermined room were selected as reference points to generate PDRM in this positioning level. Each of these reference points was labeled with its coordinates on the complete floor grid. As in the case of RDRM, only the available APs in the pre-determined floor and the existing walls were used to generate PDRM. KNN with k = 1 was used as a positioning algorithm retrieved the coordinates of the point that best matched with each testing point. In addition, Euclidian Distance geometry was used to compute the distance error between the selected RM point and the testing point. 

**Figure 13:**
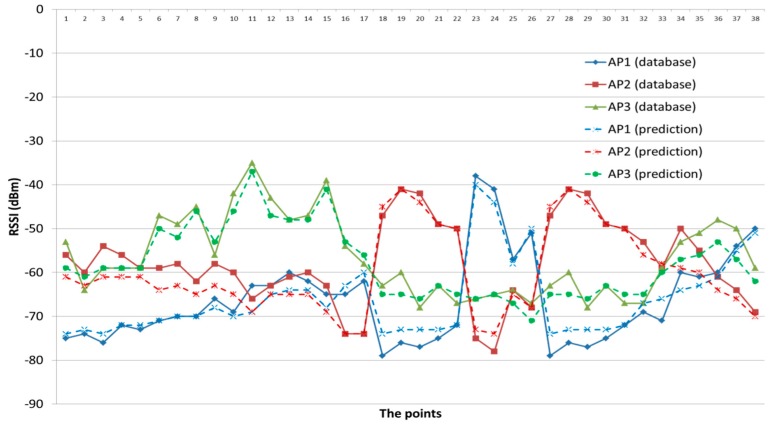
The database and prediction of RSSI.

**Figure 14:**
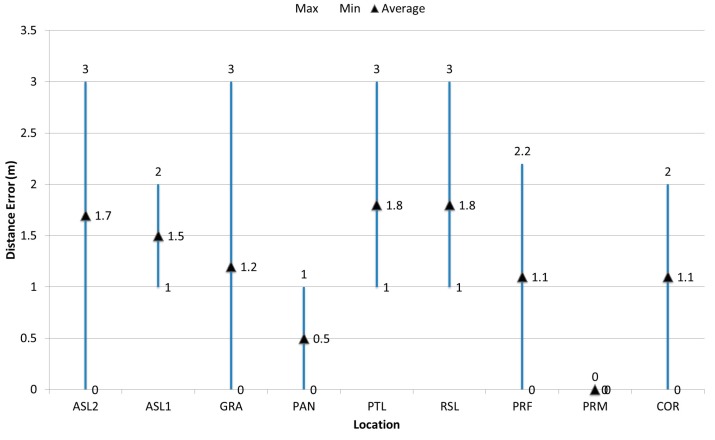
Average distance error per the test-bed layout.

Figure 13 shows the database and prediction of RSSI, and Figure 14 shows the important statistical values, such as the minimum; maximum and the average, of the computed distance error in each room or location. The minimum distance error did not exceed 1 m. The maximum distance error (3 m) is considered relatively small in comparison with other works such as [55]. On the other hand, the achieved average distance error gave very interesting results since the achieved maximum average was 1.8 m in each room or location. This promising result was achieved due to three different reasons: (1) the hierarchal positioning levels which as adopted in the proposed DIPS; (2) the small size of the generated PDRM of the pre-determined room; (3) the accurate determination of the parameters of the path loss model.

The validation process showed that the generated DRM achieved 98% and 92% of positioning accuracy for floor and room positioning respectively. For point localization, the validation process showed that the generated DRM achieved 1.2 m distance error. In comparison with the most recent related works as shown in Table 1 and Table 2, the automatically generated DRM achieved the highest positioning result accuracy. It is hard to infer the exact testing environment from previous works. Hence we only listed the results (accuracy, distance error) from the most related works and compared it with our proposed method. 

### 4.2. Solution of Mobile Devices Heterogeneity

**Figure 15:**
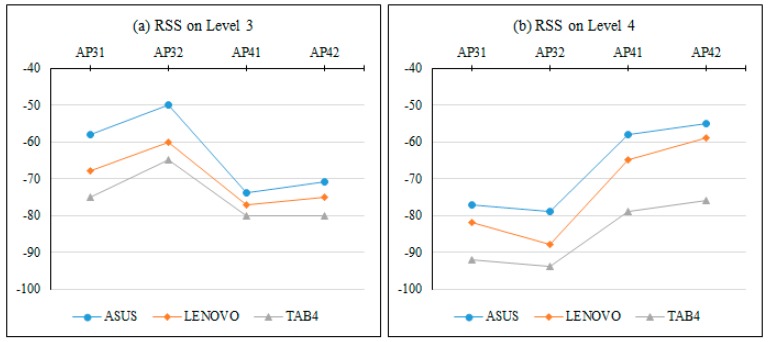
The calibrated RSS differences due to MD heterogeneity in different floors.

The result of the experiments explained in Section 3.2 can be seen in Figure 15. It shows line charts which represent the calibrated RSS tuples over two different floors. Hence, over all the calibrated RSS tuples there is a pattern can be exposed. That pattern exposed that the highest RSS value in the calibrated RSS tuple came from the nearest AP, and the lowest RSS value came from the farthest AP and so on regardless of the used MD.

**Figure 16:**
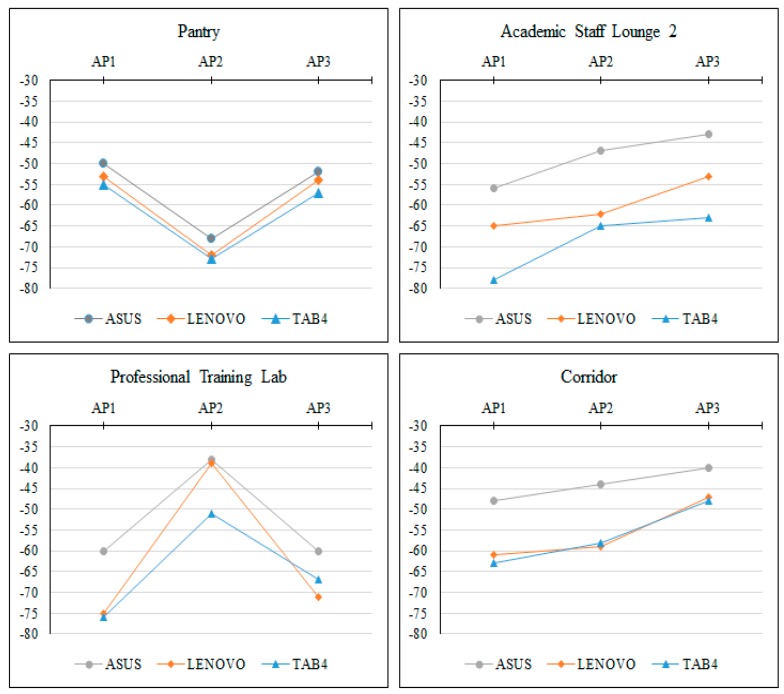
The calibrated RSS differences over four different reference points.

In order to provide an overview of RSS differences due to different MDs, a line chart based on the absolute test set was drawn as shown in Figure 16. The chart shows that the absolute calibrated RSS by the different mobile devices from the available access points (AP1, AP2, AP3) on four different reference points in different rooms. These rooms are Pantry, Academic Staff Lounge 2, Professional Training Lab and Corridor. The line charts showed that there is a general trend or phenomenon in representing the absolute RSS of each MD. This trend exposed that the highest RSS value in the received signal tuple came from the nearest access point, and the lowest RSS value came from the farthest access point and so on regardless of the used MD. 

**Figure 17:**
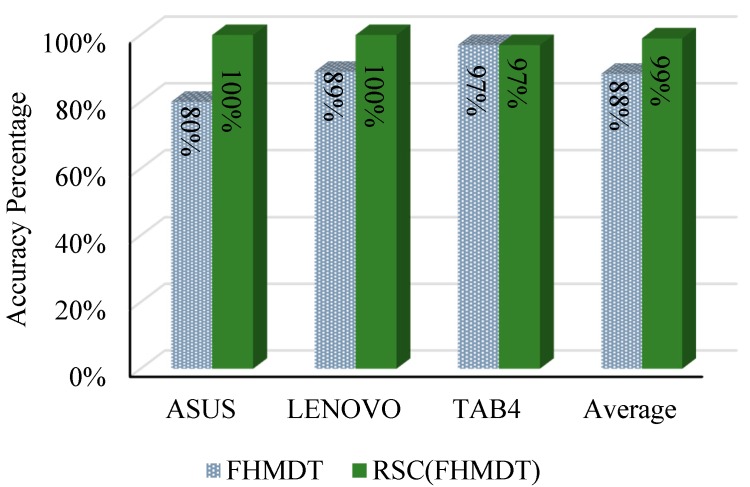
Floor positioning accuracy percentage before and after applying RSC, FHMDT and RSC (FHMDT).

The obtained positioning accuracy before and after applying RSC is presented in Figure 17. These results show that heterogeneous MDs provided different accuracy positioning because each of them have different RSS tuple or pattern, as shown in the previous section, and this tuple differs from the automatically generated pattern for the same testing points. The mean of all the achieved positioning results was 88% for floor positioning, after applying RSC on 8FDRM and HMDT sets the overall average of achieved positioning accuracy increased by 11%. 

**Figure 18:**
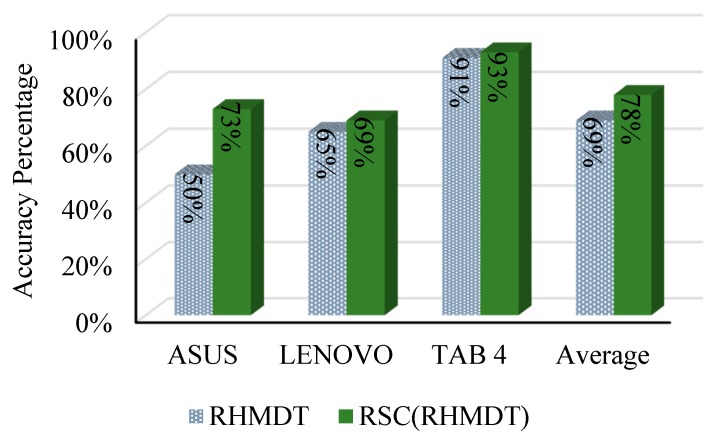
Room positioning accuracy percentage before and after applying RSC, RHMDT and RSC (RHMDT).

Room HMDT (RHMDT) was used to validate the effect of RSC on room positioning level. Figure 18 summarizes the obtained positioning accuracy before and after applying RSC on RHMDT. The graph shows that applying RSC on RHMDT increased the positioning accuracy of the ASUS MD by 23% to reach 75%, increased the positioning accuracy of the LENOVO MD by 4% to reach 69% and increased the positioning accuracy of the TAB4 MD by 2% to reach 93%. 

Point HMDT (PHMDT) was used to validate the effect of RSC on point positioning level. The distance error was adopted as validation metric in this validation level and it was measured before and after applying RSC. Paired-samples *T*-test was adopted to show the significance before and after applying RSC on point positioning level. Paired-samples *t*-test used when we need to compare the mean for the same group of sample on two different cases, or when we have matched pairs. The significance (2-tailed) value of the SPSS application of the Paired-samples *T*-test for the obtained distance error based on PHMDT and RSC (PHMDT) is 0.000, which is lower than 0.05, shows statistically there is a significant difference in applying RSC for point positioning level. 

Hyperbolic Location Fingerprinting (HLF) was proposed by Kjaergaard [41] by replace the absolute $RSS_{i}$ in both RM and the calibrated RSS tuple, by the ratio between each pair of the received signal. Hossain [61] proposed to replace the absolute $RSS_{i}$ in both RM and the calibrated RSS tuple, by the signal strength difference (SSD) values between each pairs of the received signal n-tuple. Zheng [14] proposed weight-RSS (w-RSS) by combine the absolute RSS and its relation, i.e., it computed the distance between the online RSS tuple and the offline one by involving its weight. 

RSC and the selected related works were tested based on the generated RDRM for the selected testbed and RHMDT set. Table 3 provides the obtained positioning accuracy based on the HLF, SSD, wRSS and RSC methods. This comparison shows RSC as the best base method to handle the device heterogeneity. 

### 4.3. People Presence Effect

**Figure 19:**
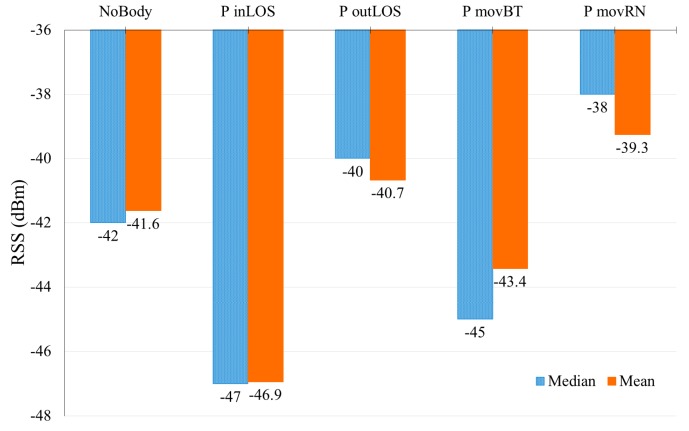
PPE in HLOS RSS centroid values.

Figure 19 shows the minimum, maximum and RSS’s centroid values for all scenarios, based on the experiment explained in Section 3.3.2. It easily shows the negative effect of peoples’ presence on the received signal strength. For the first scenario (NoBody), RSS fluctuates maximally by −4 dBm. For the second scenario (P inLOS), where one person was in the room and he was not in the LOS between AP and MD, RSS fluctuation range increased to reach −6 dBm. In the P outLOS scenario, one person was positioned in LOS between AP and MD, the fluctuation range of RSS was equal to −5 dBm. The effect of a moving person on the LOS is presented in the P movBT scenario where the RSS fluctuation range increased to reach −15 dBm. This value occurs because the person moved between AP and MD and in this movement when the person became close to MD his effect increased and it decreased as he was further away. For example, the minimum RSS value (−50 dBm) calibrated while the person was too close to the MD and the maximum RSS value (−36 dBm) calibrated while the person was at a further distance from the MD because the effect of multipath strengthened the RSS along the distance between the person and MD. Finally, the last scenario (P movRN) in this experiment shows that the randomly moved person affected the calibrated RSS values which ranged from −46 dBm to −36 dBm. Thus, peoples’ presence in HLOS affected the calibrated RSS and this effect reached its maximum value when the person stands between the AP and MD as in P inLOS.

**Figure 20:**
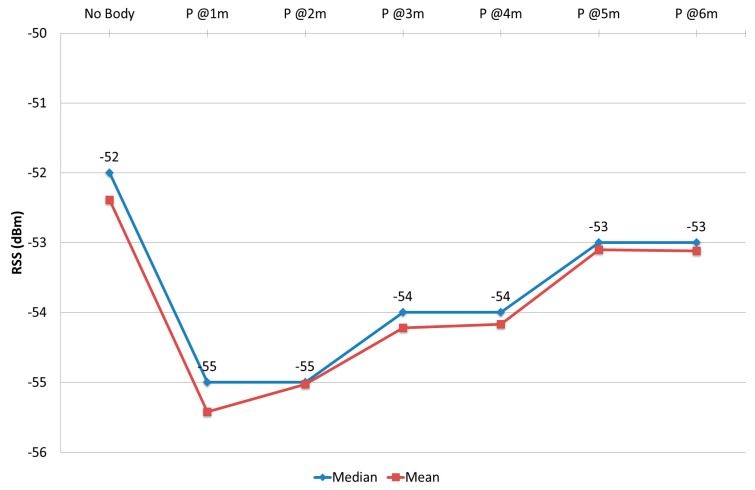
PPE in DLOS.

The result of the experiment described in Section 3.3.3 can be shown at Figure 20. It shows that the median RSS value reached its lowest value, which is −55 dBm, when the person was positioned at the closest point at 1 m from MD with −3 dBm of difference from the control case “No Body”.

In addition, this RSS value recovered its strength gradually as the person moved further away from the MD to reach −53 dBm when that person was positioned at 6 m from the MD. With the assumption that a small change (−1 dBm) will not be considered, RSS completely recovered its strength after 4 m. Furthermore, this result supports the results of the previous section. In general, these values were obtained because when the person is close to the MD his or her body hinders the direct signal towards the MD and prevents the multipath signals from strengthening the main signal. This means more variation in the received signal, so that the distribution values were high. When a person is positioned close to MD in DLOS with 1 m distance between them, the calibrated RSS declined by −2 dBm to −3 dBm. 

**Figure 21:**
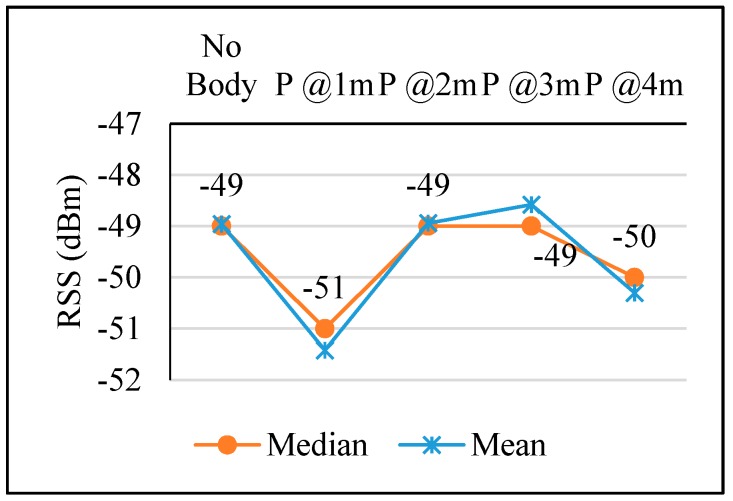
PPE in VLOS case 1.

**Figure 22:**
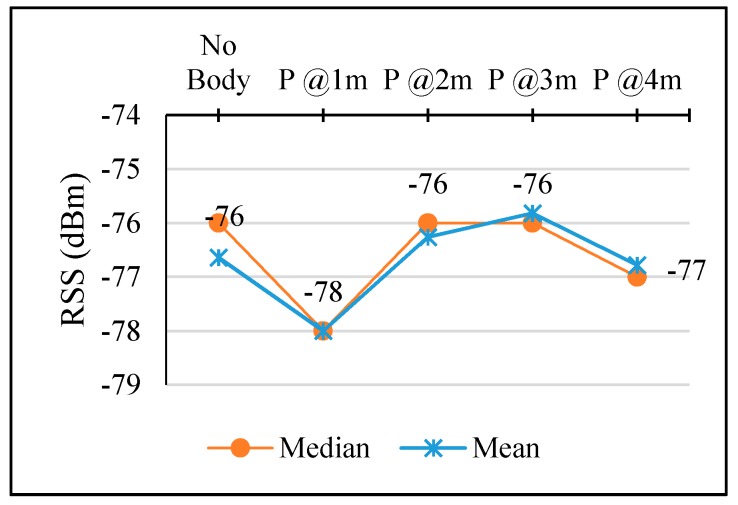
PPE in VLOS case 2.

Figure 21 and Figure 22 show the result of the experiment in Section 3.3.4. RSS reached its lowest value, −51 dBm and −78 dBm respectively, when the person was located at 1 m of distance from the MD within the virtual line between the AP and MD. Then the RSS value started to recover its strength gradually as the person was further away. This is because when the person was located at a close point with respect to the MD between the AP and MD, his/her body hinders the incoming signals and diffracts them. Hence, the MD can receive the reflected signals after that person’s body and they must be lower than the expected ones.

The obtained results from all previous experiments, which aimed to show PPE on RSS in HLOS; DLOS and VLOS, showed that same trend. This trend is that RSS was at its lowest values as the person was located close to the MD between the AP and MD. Then RSS started to recover its strength gradually as the person was further away from the MD. In addition, the distributed probability values of the calibrated RSS reached it maximum values as the person was located close to the MD between the AP and MD. Then these distribution values started to decline slowly as the person was located in further distance from MD.

**Figure 23:**
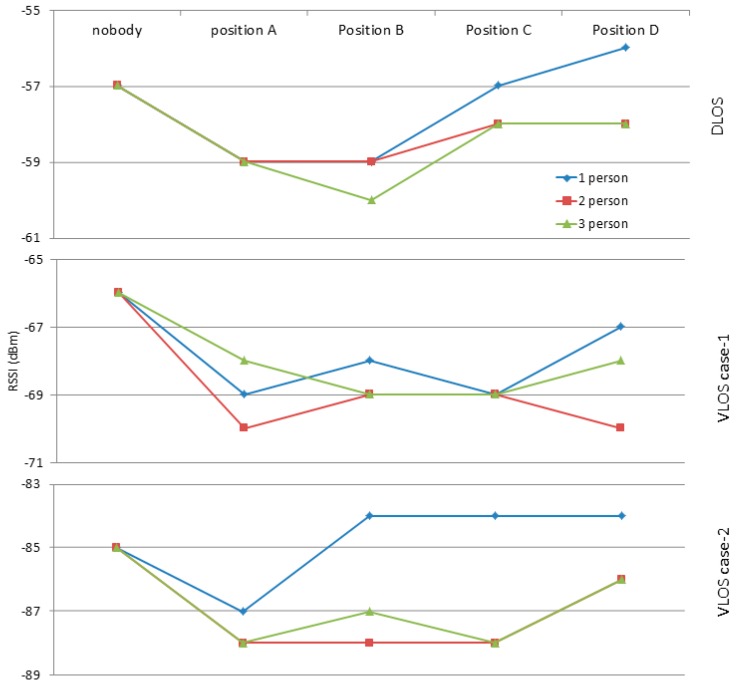
People presence effect on RSS and influence distance.

Figure 23 shows the result of experiments to define PPE Influence Distance as described in Section 3.3.5. There are three cases (DLOS, VLOS case-1, and VLOS case-2) in these experiments, and each case involves 1, 2, and 3 persons. The position of the person can be seen in Table 4.

In comparison to the NoBody case, the person presence affected the calibrated RSS as the person located at distances less than the influence distance. In addition, the calibrated RSS recovered its strength even with the presence of a person at distances larger than the influence distance. This means when the person could not hinder the LOS between AP and MD, the calibrated RSS recovered it strength as there is no any obstacle. In addition, PAF will be adopted to be −2 dBm as the most frequent value as a result of PP. 

**Figure 24:**
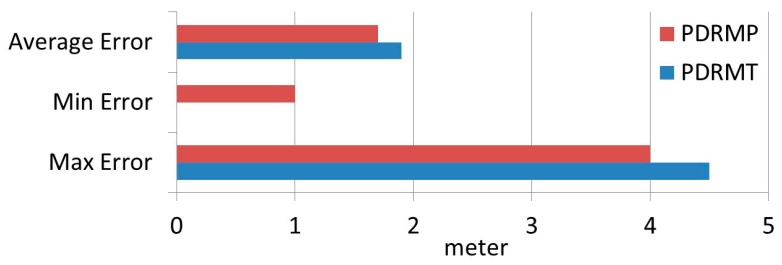
Distance error based PDRM with and without considering PPE.

Figure 24 shows the obtained distance error based on PDRMT and PDRMP. The average distance error shows that there is a slight enhancement (0.2 m) on the obtained positioning result. This enhancement is consider important for two reasons. First, this slight enhancement includes more than 50% of the obtained results less or equal to 1 m; second, based on our literature review, this is the first time in which PPE was included in IPS. 

## 5. Conclusions

In this research study, an adaptive indoor positioning model (DIPS) based on WLAN fingerprinting for dynamic and multi-floor environments was designed and validated. DIPS contained three main components which are: Dynamic RM Generator (DRMG), RSS Certainty (RSC) and People Locations component which was added to consider the influence of peoples’ presence as PAF in the proposed path loss model to generate a realistic DRM. DRMG was used to overcome the manual RM calibration issues as well as to overcome the effect of the environmental change on the obtained positioning accuracy. DRMG was developed based on the modified path loss model and relied on the environment layout description. The experimental work and the benchmarking showed that DIPS, DRMG, provided high positioning accuracy reaching 100% for floor positioning, 93% for room positioning and 1.2 m of dstance error for point positioning. Furthermore, this promising positioning results did not require any extra devices in the targeted environment.

RSC is presented as a robust solution for the MD heterogeneity problem. The experimental work showed that RSC enhanced the positioning accuracy significantly (99% for floor positioning accuracy, 78% for room positioning accuracy, and 1.9 m for distance error). The significance of positioning enhancement was proved statistically by using the paired-sample *T*-test. Furthermore, RSC was benchmarked against some of the most recent related works, such as Weighted RSS (wRSS), Hyper Location Fingerprint (HLF) and Signal Strength Difference (SSD). The benchmarking results showed that RSC achieved the highest positioning results with enhancements ranging from 15% to 19%.

PPE to RSS within the influence distance (PPID), in both DLOS and VLOS cases, was modeled by −2 dBm for the person closest to the MD. PPE was integrated into DRMG to generate DRM for point positioning level. The experimental work was conducted in a free space environment to validate this integration empirically. The obtained results showed that considering this influence as signal attenuation factor in generating PDRM reduced the distance error by 0.2 m to reach 1.7 m.

Some future work directions are finding an automatic way to detect and identify any environmental change occurrence, and accordingly update the environment layout description automatically. Validating the proposed DIPS accuracy on different positioning algorithm such as Weighted KNN (WKNN) and Learning Vector Quantization (LVQ) algorithms. Validating the scalability and robustness by testing the proposed DIPS in crowded multi-floor environments.

## Figures and Tables

**Table 1:** Related work floor positioning techniques and accuracy rate.

Authors	Used Technique	Accuracy
Shi [56]	Path loss model with Feedback analysis	Up to 100%
Campos [57]	Kohenon and Backpropagation Neural Network	91–97%
Gupta [58]	Radio propagation model, maximum likelihood and pressure sensor	Up to 100%
Maneerat [59]	WSN with confidence interval	Up to 100%
Sun [10]	Fisher’s Linear Discriminant and weighted KNN	94%
The Proposed Model	Path loss model + KNN	93–100%

**Table 2:** Related work floor positioning techniques and distance error.

Study	The Method Used	Distance Error (m)
Bahl [18]	Manual Radio Map + KNN	2.5
Hung-Huan [31]	Path Loss model + Triangulation	1.6
Vahidnia [60]	Manual Radio Map + BPM	1.4
Sun [10]	Manual Radio Map + Weighted KNN	1.2
The Proposed Model	Path Loss Model + KNN	1.2

**Table 3:** Positioning results based on different calibration-free techniques.

Calibration-Free Techniques	Mobile Devices			Positioning Average
	ASUS	LENOVO	TAB4	
w-RSS [14]	38%	69%	69%	59%
HLF [41]	58%	73%	54%	62%
SSD [61]	65%	65%	58%	63%
RSC	73%	69%	93%	78%

**Table 4:** People position in experiments to define PPE influence distance.

Number of Persons	Distance from MD (m)			
	Position A	Position B	Position C	Position D
1 person	1	2	3	4
2 person	1 and 2	1 and 3	1 and 4	2 and 3
3 person	1, 2, and 3	1, 2, and 4	1, 3 and 4	2, 3 and 4
